# Imperfect diagnosis: The truncated legacies of Zika testing

**DOI:** 10.1177/03063127211035492

**Published:** 2021-08-31

**Authors:** Koichi Kameda, Ann H Kelly, Javier Lezaun, Ilana Löwy

**Affiliations:** 1 Institute for Research and Innovation in Society (IFRIS), Paris, France; 2 Department of Global Health and Social Medicine, King’s College London, London, UK; 3 Institute for Science, Innovation and Society, University of Oxford, Oxford, UK; 4 INSERM, CERMES 3, Villejuif, France

**Keywords:** Zika, diagnostics, global health, emergency R&D, Brazil

## Abstract

When the Zika virus burst onto the international scene in the second half of 2015, the development of diagnostic tools was seen as an urgent global health priority. Diagnostic capacity was restricted to a small number of reference laboratories, and none of the few available molecular or serological tests had been validated for extensive use in an outbreak setting. In the early weeks of the crisis, key funders stepped in to accelerate research and development efforts, and the WHO took responsibility for steering diagnostic standardization, a role it had successfully played during the West Africa Ebola virus outbreak. Yet when the WHO declared the end of the Zika Public Health Emergency of International Concern in November 2016, diagnostic capacity remained patchy, and few tools were available at the scale required in the countries that bore the brunt of the epidemic, particularly Brazil. This article analyses the limited impact of global R&D efforts on the availability of Zika diagnostic options where they were most needed and for those most vulnerable: women who might have been exposed to the virus during their pregnancy and children born with suspected congenital Zika syndrome. The truncated legacies of testing during the Zika crisis reveal some of the fault lines in the global health enterprise, particularly the limits of ‘emergency R&D’ to operate in geopolitical contexts that do not conform to the ideal type of a humanitarian crisis, or to tackle technical issues that are inextricably linked to domestic struggles over the scope and distribution of biological citizenship. Diagnostic shortcomings, we argue, lie at the heart of the stunning transformation, in less than two years, in the status of Zika: from international public health emergency to neglected disease.

In the early autumn of 2015, Zika burst onto the international scene as a sudden and deeply alarming public health threat. The Zika virus (ZIKV) was first identified in 1947, but it had attracted little scientific attention until an outbreak in Brazil’s Northeast was associated with an unusual cluster of microcephaly among newborns. The possibility that severe foetal abnormalities could be caused by a virus transmitted by a mosquito species (*Aedes aegypti*) that is endemic across tropical and subtropical regions prompted an international crisis. As the Brazilian government struggled to mount an effective response in what was quickly becoming the epicentre of a global outbreak, key gaps in virological, pathological and epidemiological knowledge became the focus of urgent attention. On 1 February 2016, the WHO declared a Public Health Emergency of International Concern (PHEIC), with the explicit goal of addressing these epistemic and technical deficits (Heymann et al., 2016; Kelly et al., 2020). Without a rapid and significant investment in Zika research and development (R&D), the options for intervening in the epidemic, let alone bringing it to a halt, appeared limited.

The most urgent priority was the development of reliable ways of detecting Zika virus infection. Accurate differential diagnosis is a *sine qua non* for outbreak control: It enables the isolation and (when possible) treatment of patients, underpins the production of epidemiological knowledge and is a precondition for the development of effective therapeutics. Zika, however, presented from the start numerous diagnostic challenges. The vast majority of cases appeared to be asymptomatic, and when symptoms appeared they were generally mild and self-limiting, with patients reporting nonspecific aches, skins rashes, low fever and general fatigue. Several of these symptoms resembled those associated with dengue – early reports, in fact, often described the condition as a ‘mild’ form of dengue – another virus transmitted by Aedes mosquitoes that is endemic across the region.

Diagnostic challenges went far beyond clinical case detection. Early studies suggested low and brief viraemia, leaving a short window – less than a week after the onset of clinical symptoms – for the detection of viral genetic material. As far as the identification of antibodies was concerned, structural similarities between ZIKV and other flaviviruses created a high potential for cross-reactivity in serological tests. In addition to dengue, which presents diagnostic challenges of its own, chikungunya had been circulating in Brazil since at least 2014, and yellow fever was re-entering the periphery or large urban areas. Zika’s teratogenic potential gave these technical hurdles an acute moral charge. Pregnant women who feared they might have been infected were desperate for immediate and accurate results. The fact that in many of the countries most affected by the outbreak, including Brazil, abortion is illegal and often criminalized entangled the question of diagnosis in fierce disputes over sexual and reproductive rights.

Diagnosis – from *dia/gnosis*, or knowing apart – is expected to produce certitude and enable effective health interventions, but it requires a complex coordination of epistemic objects, analytical techniques and moral concerns. The Zika emergency of 2015–2017 offers an opportunity to explore the pragmatics of that coordination over the course of a global health emergency, when uncertainties cut across multiple domains of clinical, epidemiological and biosecurity practice (Wilkinson, 2017). Drawing together an analysis of interviews with key actors involved in diagnostic R&D efforts during the Zika emergency and a critical review of the international and national scientific, policy and grey literature, we trace the shifting contours of diagnostic priorities through the course of the Zika response, attending in particular to the ultimately unsuccessful alignment of global research efforts and domestic needs in Brazil.^1^

The paper is structured in five parts. In the first section we briefly revisit some insights from the social scientific literature on diagnostics, particularly Ludwik Fleck’s understanding of diagnostic stabilization. We go on to explore the WHO’s efforts to accelerate the global development of Zika tests, and the significant obstacles these efforts encountered. The limits that inadequate diagnostic capacity placed on the production of epidemiological knowledge forms the focus of the third section, where we consider how limited access to molecular and serological testing shaped public health intelligence in Brazil. Next we explore the process by which cases of congenital Zika syndrome (CZS) – the designation for newborns whose neurodevelopmental impairment was officially linked to prenatal ZIKV infection – were confirmed, and the impact that the dearth of actionable medical knowledge had for the mothers of children born with microcephaly. In the conclusion, we reflect on the truncated legacies of testing during the Zika emergency, and discuss the significance of this experience for future conceptualizations of effective global health action.

## Diagnostic coordinations

Diagnosis is the cornerstone of modern medicine. Until the mid-nineteenth century, human pathologies were seen as situated, fluid entities: events shaped by the particularities of a singular life and originating in the unique interaction of the patient’s constitution, environment, life-style and life-course. This understanding of disease as a mutable phenomenon, inseparably linked to bodies and the places they inhabit, changed radically with the development of bacteriology. Following the discovery of several causal agents of infection, diseases were slowly re-conceptualized as distinct entities, existing outside the unique manifestations of ill health in a given individual (Rosenberg, 2002).

From this moment on, the first task of the physician was to correctly diagnose the ‘specific disease’ that afflicted the patient. The standardization of disease categories presupposes, however, the stabilization of diagnostic tools and practices. The pioneer of social studies of scientific knowledge, Ludwik Fleck, provides crucial insights into the pragmatic aspect of these processes of stabilization. Speaking from the unique standpoint of a microbiologist and a public health expert, Fleck advances the argument that the goals of any given professional group will shape the scientific facts they produce. Scientists investigating a new disease and epidemiologists concerned with limiting its spread are likely produce divergent data on the prevalence of a transmissible pathology: Researchers seeking to elucidate the specific biological mechanism of host-pathogen interaction will tend to exclude all borderline cases, while epidemiologists intent on halting an outbreak will be inclined to include them (Fleck, 1986[1929]).

Fleck develops this idea further in his *Genesis and Development of a Scientific Fact*, a detailed study of the development of the Wassermann reaction, a serological test for syphilis (Fleck, 1979[1935]). In the early twentieth century, experts came to agree that the term ‘syphilis’ described an infection caused by the microorganism *Treponema pallidum*, and this definition opened the way to diagnosing syphilis by detecting anti-Treponema antibodies in the patient’s blood. The development of a diagnostic test proved elusive, however. The Wassermann reaction was exceedingly difficult to stabilize – it included two distinct steps, each involving several biological reagents, and any variation in any of the reagents or in the conditions of laboratory work appeared to affect the final result. A first test believed to detect antibodies specific to *Treponema pallidum* was later re-defined as visualizing poorly understood ‘changes in the blood’ of Treponema-infected individuals.

How, despite these challenges, the Wassermann reaction came to be accepted as the appropriate way to provide a differential diagnosis of syphilis in clinical settings is the core of Fleck’s analysis. Fleck argues that the process through which the reaction was endowed with the ability to produce medical facts can only be understood with reference to the uses the diagnostic tool was meant to fulfill, and the political weight those uses carried at the time. Syphilis was perceived during the interwar period as an urgent public health problem, threatening not only present but also future generations. This view of syphilis as a ‘global menace’ led to a massive investment of money, labour and time in the transformation of a highly problematic technology into one that could be effectively harnessed to fulfil an imperative public health goal. Through a series of international conferences convened by the League of Nations, experts from around the world achieved a degree of calibration and homogenization that made the Wassermann reaction, despite all its imperfections, the ‘right tool for the job’ (Löwy, 2004). That collective effort of scientific and diplomatic coordination, propelled by a sense of moral urgency, thus engendered a new scientific fact: A positive result in the Wassermann reaction was equated with an active infection by *Treponema pallidum*. This was quickly followed by the rapid diffusion of the test in key populations, such as army recruits or pregnant women, and by the introduction in many countries of obligatory pre-nuptial screening for syphilis. A scientific fact thus became a legal fact, with far-reaching implications for patients’ civil rights and life-courses.

Fleck’s analysis bears special significance today, when the rise of ‘global health’ has expanded and transformed the role of diagnostics in the control of transmissible diseases. The need to extend diagnosis from the hospital or the laboratory to the rural clinic and other ‘resource constrained’ settings has prompted an explosion in innovation, including a new generation of rapid, highly portable, ‘point-of-care’ tests. This ‘testing revolution’ (Street, 2018) comes hand in hand with organizational models and funding mechanisms designed to reconcile humanitarian principles with profit incentives, and to align action across highly heterogeneous local, national and global scales, making the sort of techno-political coordination Fleck described particularly salient and fraught (Engel, 2020; see also Lezaun, 2018; Nading, 2015).

## A ‘global’ race for new Zika diagnostics

Following the declaration of the Zika PHEIC on 1 February 2016, the WHO initiated an emergency R&D plan to address the urgent need for preventative, diagnostic and therapeutic measures. The WHO had tested this sort of ‘accelerated R&D strategy’ during the West African Ebola virus outbreak of 2014–2015, which by the time Zika came to global health attention had yet to be fully contained. The rapid spread of the Ebola virus, the mortality associated with the disease, and the lack of therapeutic options had prompted a radical revision of the norms and standards of medical research practice, and led to the introduction of a new set of investigative protocols. In an effort to compress innovation timelines, the WHO launched a new Emergency Use Assessment and Listing procedure (EUAL), a fast-tracked regulatory pathway designed to facilitate the procurement of unproven but promising technologies, allowing access to these tools while evidence of their effectiveness was still being collected.^2^

In the case of Ebola, the effort to accelerate R&D was, broadly considered, successful. Biomedical development timelines, typically measured in years, were radically shortened, enabling vaccine, therapeutic and diagnostic candidates to reach affected populations within a matter of months. At a time when the sluggishness of the WHO’s response to the outbreak was the subject of excoriating criticism, these investigative successes salvaged the organization’s reputation (Kelly, 2018). Building on this experience, the WHO went on to publish its ‘R&D Blueprint For Action to Prevent Epidemics’, laying out a strategic framework for the ‘rapid activation of R&D activities’ and the establishment of a ‘research-enabling environment’ in times of public health emergency (World Health Organization [WHO], 2016).

Following hard on the heels of the Ebola outbreak, the Zika crisis was, in the words of WHO Assistant Director-General for Health Systems and Innovation Marie-Paule Kieny, ‘an important test case’ for the R&D blueprint.^3^ Immediately after the PHEIC declaration, the WHO commissioned the development of international standards for ZIKV detection and convened a consultation process with members of the scientific community, regulatory agencies, funders and public health organizations ‘to expedite the development of the products required for a rapid, robust response to Zika virus’ (WHO, 2016: 8). At a first meeting in Geneva in early March 2016, the field of Zika diagnostics was described as promisingly ‘busy’, with at least eight companies already offering ‘commercially available’ molecular tests and many more interested in entering the market. A few days earlier, the United States had activated its own provisions for emergency use authorization, and the Food and Drug Administration had given the green light to the IgM antibody capture ELISA test that the US Centers for Disease Control and Prevention (CDC) had developed in the aftermath of the 2007 Zika outbreak in Yap Island, Micronesia. The FDA also provided emergency use authorization for the CDC’s in-house RT-PCR assay.

The impression conveyed in these early meetings was that ‘the product development community has responded energetically to the need for these resources, and [that] the pipeline is expanding rapidly’ (WHO, 2016: 8). Yet much remained to be done to translate this initial burst of energy into a set of effective tools. As a participant in the first WHO-convened consultation on Zika R&D noted, ‘outbreaks are unsustainable markets,’ as profits are tied to the evolution of the epidemic, and this is particularly true in the field of diagnostics, where few developers have the staying power to weather a long period of uncertainty over the commercial prospects of still unproven products (WHO, 2016). In this context, the WHO saw its role as bringing stability and predictability to a volatile market by defining the technical parameters of acceptable diagnostics, so that manufacturers could invest the necessary resources with the knowledge that, if their products met these criteria, the WHO would fast-track their assessment and issue an emergency listing to allow their procurement by UN agencies and national governments.

After a series of discussions with stakeholders, the WHO and its partner organizations UNICEF and PAHO issued two ‘target product profiles’ (TPPs) for Zika diagnostics, one for the detection of active infection and a second one for the detection of past infection (Chua et al., 2017). These targets were ambitious. The TPP for active ZIKV infection specified a minimum sensitivity of 95% and a minimum specificity of 98%, and noted that the tests should ideally be usable in primary health facilities and by health workers with minimal training; the TPP for detection of past Zika infection was very similar, except for a slightly lower specificity threshold (95%).

Following the publication of the TPPs in April 2016, thirty-three applications were submitted to the EUAL from established manufactures, biotech start-ups and academic laboratories. By the time the PHEIC was lifted in November of that year, however, only two of these applications had resulted in products listed for emergency procurement. Both were molecular RT-PCR methods for the detection of ZIKV ribonucleic acid (RNA): a multiplex test for the detection of Zika, dengue and chikungunya virus RNA, developed by the South Korean firm Bioneer Corporation, and a RT-PCR kit manufactured by the German firm altona Diagnostics. No antibody or rapid diagnostic was listed as eligible for WHO procurement, and many of the firms that had expressed an interest in developing new products quickly exited the field as soon as the WHO declared the end of the emergency.

The reasons for the paucity of new products were multiple. In contrast to the Ebola virus, which had been subject to extensive research as a high-priority bioterrorism agent for at least two decades, there was very little pre-existing scientific expertise on key aspects of ZIKV’s viraemia, infection kinetics or immune response. Diagnostic development had to start from scratch, with no international reference materials, harmonized assays or well-characterized reagents to validate products. The EUAL mechanism had been designed to address situations under which ‘the community may be willing to tolerate less certainty about the performance and safety of the products’ (WHO, 2015: 1). It was less well suited to address the challenges presented by the Zika emergency, where the very principles of diagnosis were still uncertain and an entirely new testing infrastructure had to be built. The lack of expertise and technical capacity was evident in the low quality of many of the applications for emergency use listing. ‘Overall’, the WHO noted in a review of the process, ‘the submitted technical documentation has been poor, necessitating supplementary laboratory evaluation by WHO.’ Very few laboratories, however, were capable of undertaking these supplementary evaluations, and this was particularly true in the countries that were bearing the brunt of the epidemic.^4^

A key factor in the creation of a new Zika diagnostic infrastructure was the availability of specimens and samples from patients infected with the virus. The difficulty developers encountered when they sought to gain access to those materials underscores another critical difference with the Ebola emergency: the complex relationship between national and international research contexts. In 2014, the Ebola virus had erupted in one of the poorest parts of the world, in countries whose public health systems had been devastated by decades of civil war. In addition to little or no laboratory infrastructure, Guinea, Liberia and Sierra Leone suffered from a dearth of expertise on analytical validation and large-scale clinical research. With no in-country facilities able to confirm an Ebola case, the development of reliable diagnostics was seen, at least at the start of the outbreak, as an absolute priority, and in the absence of strong local academic support or regulatory oversight the task fell to international actors (divided largely along former colonial lines). The emergency also provided ethical cover to bypass overwhelmed national authorities – tens of thousands of samples were exported abroad, often under the pretext of preserving precious, non-renewable resources that would be needed to tackle future outbreaks of the virus. This breach of sovereignty would prompt considerable outrage in years to come, but in the meantime it allowed a wide distribution of biological materials to public and private laboratories in high-income countries (WHO, 2015).

The geopolitics of the Zika outbreak were very different. Brazil, the country at the centre of the emergency, has an advanced biomedical research infrastructure and a famed universal health care system. Historically, the state’s many successes in public health have been linked to an activist stance in relation to the development of ‘national’ health technologies (Benchimol, 2001; Cassier and Correa, 2018; Hochman, 1999; Kropf, 2009). Brazil’s approach to public health has, moreover, a clear international dimension – it represents an alternative, powerful vision for ‘global health,’ as became evident when, at the turn of the century, the country played a leading role in the struggle for universal access to antiretroviral treatments. By the time the Zika emergency hit, these global aspirations had been dented by domestic economic and political crises, yet Brazil retained three elements that were bound to play a crucial role in the response to the Zika crisis: a complex regulatory system for novel medical products, a series of state-funded institutions with experience in the development of novel diagnostics, and a tradition of asserting sovereign rights over biological resources. The Zika emergency in fact coincided with the passing of a new national law according to which any microorganism isolated in Brazil was part of the country’s ‘genetic heritage’ (*patrimônio genético).* Signed by President Rousseff on 20 May 2015, the law introduced a new set of conditions for the international shipment of biological samples. Yet it only entered into force on 17 November 2016, leaving Brazilian research institutions in a legal limbo for much of the emergency and complicating the transfer of materials to foreign laboratories.

Brazil’s restrictions on the international transfer of biological materials are not dissimilar from those enforced by many developed countries, and are motivated by the well-founded fear that local resources could be used to generate commercial products that will be unaffordable to its national healthcare system. The restrictions had nevertheless a significant impact on international efforts to develop new diagnostics. Throughout the Zika emergency, and despite high-level diplomatic entreaties from the WHO, the US government and others, the Brazilian government remained reluctant to expedite the international transfer of biological samples or specimens. As the coordinator of the WHO’s programme to produce an international standard for Zika antibody, noted: ‘We soon learnt that sera/plasma and viruses from Brazil were off the table’ (Page, 2019).

The result was that public health laboratories around the world, including national reference facilities, were forced to rely on materials collected during previous Zika outbreaks, or to procure new samples through ad hoc arrangements with private Brazilian laboratories. ‘It’s not going via official government channels’, a prominent European virologist noted in early 2016. ‘Our source is simply the rich people [in Brazil] who want a diagnosis’ (Cheng et al., 2016). In the United States, government agencies developed an ‘emergency use simple letter agreement’, or EUSLA, to facilitate access to Zika-relevant biological materials, and more than 160 transfers were completed under its provisions, but the sourcing of samples remained a significant problem for US federal laboratories until cases of ZIKV infection emerged in Puerto Rico in January 2016.^5^

The limited circulation of biological materials constrained and slowed down the global product pipeline for commercial diagnostics. Even in the ‘simplified’ terms stipulated by the EUSLA, the legal requirements of material transfer and the limitations they placed on the future use of the samples presented too high a bar for many developers and involved a more extensive timeline than the WHO EUAL had intended (Halabi, 2019). Many commercial firms chose instead to use samples from international travellers who had been infected with Zika while visiting foreign countries. This allowed those firms to develop new tests fairly quickly, but complicated the calibration of assays and the design of international studies to evaluate their performance. In the case of antibody tests, it also raised doubts about the suitability of the products in countries, like Brazil, where other flaviviruses are endemic. Unlike most travellers from Europe or North America, residents in those countries would tend to have a degree of background immunity to dengue and yellow fever (as a result of prior infection or vaccination). This greatly increases the risk for cross-reactivity between antibodies triggered by different viruses, and thus reduces the specificity of any diagnostic product validated with foreign samples. Under these conditions, the global harmonization of ZIKV serology proved to be much more protracted than initially anticipated. The WHO would only issue its International Standard for anti-Zika virus antibody in October 2018, almost two years after the lifting of the PHEIC, by which time the epidemic had subsided and much of the pressure to accelerate R&D efforts had dissipated (see Koopmans et al., 2019).

Struggles over exchange and harmonization are common to most international research collaborations, but in the case of Zika they were compounded by a vexing issue that lay just beneath the surface of the myriad technical disputes. The question of what diagnosis was *for* – the ‘use-case’ of any new detection tool – was here particularly fraught because, in the absence of therapeutic or palliative options against the virus, it touched directly upon the issue of abortion. In many of the countries most affected by the outbreak, including Brazil, early termination of pregnancy is generally illegal and severely restricted. Early and accurate detection of Zika infection during pregnancy had therefore intense political implications. A representative of one of the multilateral organizations that took part in the WHO’s consultation process for Zika diagnostics recalls the acute discomfort felt at international meetings when the issue was broached:

We had this discussion with [WHO], and we said: ‘What are your plans?’ It was very sensitive, because diagnostics are used with use cases. So what is the use case of a diagnostic? If the use case is to diagnose and treat, then for Zika it was really, how can I say, a borderline issue. Because, if you want to diagnose a woman with Zika in pregnancy, what are you offering? Are you offering abortion? Are you offering a solution? This is where everybody freezes again. They don’t want to discuss this matter. We had to avoid the discussion.^6^

The question of what a pregnant woman would be able or not able to do upon receiving a Zika test result was the ‘elephant in the room’ in many such meetings, the matter that could not be addressed head-on given the intractable differences among the countries affected by the epidemic and involved in R&D efforts (Aiken et al., 2017). The inability to clarify the significance of this crucial ‘use-case’ reflects how uncomfortably issues deemed to impinge upon domestic politics, such as sexual and reproductive rights, sit within the global apparatus for outbreak response (Wenham et al., 2019). In the case of Zika, however, the matter could not be simply ignored, as it permeated all aspects of the crisis, not least the production of a reliable epidemiological picture about the extent and intensity of the crisis.

## Situational awareness

The Zika virus was first detected in Brazil in April 2015, when researchers at the Federal University of Bahia identified ZIKV RNA in blood samples collected in the nearby city of Camaçari. Official confirmation that the virus was circulating in Brazil came a few days later from the National Reference Laboratory for Arboviruses at the Instituto Evandro Chagas in Belém. Both laboratories used reverse transcription polymerase chain reaction (RT-PCR) to identify the virus, and were among the very few facilities in the country with primers for the detection of ZIKV genetic material.

For the duration of the emergency, molecular RT-PCR would remain the primary and often the only form of identification in Brazil, restricting the capacity to detect ZIKV infection to specialist laboratories. Significant knowledge gaps constrained the reliability of PCR-based diagnosis, however, and cast doubt on the sensitivity of the tests for weeks and months to come. There was virtually no information about the genomic diversity of ZIKV strains circulating in the country, and little experience of internal or external quality control for the available assays. Reagents had to be imported, or purchased from national delegations of foreign firms, often at considerable cost, placing a heavy financial burden on the central pillar of the system, the network of federal and state public health laboratories.

After the Brazilian government declared a Public Health Emergency of National Concern on 11 November, the Ministry of Health ordered that all suspected cases of ZIKV infection reported to the national Notifiable Disease Information System (the *Sistema de Informação de Agravos de Notificação*, or SINAN) be confirmed through PCR analysis. Laboratory capacity did not increase accordingly, however, and the existing infrastructure struggled to cope with the rapid increase in demand. Some private hospitals and laboratories developed their own ‘in house’ nucleic acid tests, as did some blood banks concerned with the possibility of infecting patients via transfusions, but these private endeavours remained disjointed and the data they produced generally fell outside the system of epidemiological surveillance (Kameda, 2019).

Recognizing the urgent need for more and more accessible diagnostic tools, the Brazilian regulatory agency in charge of authorizing new medical products, the *Agência Nacional de Vigilância Sanitária* (Anvisa), announced in January 2016 its willingness to fast-track applications for new Zika in-vitro diagnostics. Soon afterwards, the Ministry of Health announced a new multiplex RT-PCR capable of simultaneously detecting Zika, dengue and chikungunya viruses, developed by researchers at the Oswaldo Cruz Foundation (Fiocruz), Brazil’s flagship biomedical research institution. The bureaucratic tussle that followed this announcement illuminates some of the challenges that national diagnostic innovation encountered at the height of the emergency.

Fiocruz considered its new diagnostic an ‘in-house’ test, and as such exempt from the standard regulatory review for commercial products. The Ministry of Health had funded the development of the test and it would be responsible for its distribution to other public health laboratories, but neither the funding nor the distribution involved any ‘market’ transaction and as a consequence the test should fall outside the purview of Anvisa’s regulatory authority. Anvisa disagreed: Because the diagnostic was to be used in more than one laboratory it could not be classified as an ‘in-house’ test, and since the Ministry of Health would need to formally procure the test from Fiocruz it could only do so after Anvisa had fully evaluated the product.

Anvisa’s view prevailed, and Fiocruz’s RT-PCR test was subjected to a full review. It would eventually be authorized for commercial use, but not until December 2016, by which time the epidemic had waned. Rodrigo Stabeli, at the time Fiocruz’s Vice-President for research and reference laboratories, attributed the delay to pressure from commercial providers. ‘There must have been pressure on Anvisa to subject the Fiocruz test to the approval process. That way, companies could continue to make a profit by selling to the Ministry.’ As far as Anvisa was concerned, this was simply a matter of preserving regulatory standards. ‘We cannot have different requirements for products manufactured by private companies and by public institutions. The citizen has the right to a quality product’ (Rodrigues and Buscato, 2016).^7^

The situation with serological tests was even more convoluted. When Brazil declared a public health emergency there was no test in the country capable of detecting Zika antibodies. By the end of 2015 some reference laboratories had developed in house ELISA tests with reagents supplied by the US CDC, but capacity remained severely restricted. ‘Almost nobody had reagents. We bought them at an exorbitant price [*a peso de ouro*] – a little bit of nothing that didn’t work.’^8^ In April 2016 Anvisa authorized the first serological assays, ELISA tests for acute (IgM) and convalescent (IgG) antibodies developed by the German firm EUROIMMUN. Over the following months, this became one of the few commercial products available in Brazil, but its price (around US$33 per unit) made it unsuitable for widespread adoption in the public healthcare system, or for use in the sort of large seroprevalence studies necessary to determine the prevalence of infection in the population.

Efforts to develop a cheaper public alternative appeared to have met with success in late May 2016, when Anvisa approved a rapid antibody test developed by the Bahia Foundation for Scientific Research and Technological Development (Bahiafarma), a research organization affiliated with the Department of Health in the State of Bahia. The new tool was presented as the first ‘national’ serologic test (although it relied on technology licensed from the South Korean firm Genbody), and as a ‘game changer’ in the provision of diagnostic services, since it delivered a result in twenty minutes and was portable enough to be used in primary healthcare facilities. The Bahiafarma test was soon adopted as the standard serology tool in the national Unified Health System (*Sistema Único de Saúde*), and in October 2016 the Ministry of Health ordered around 3.5 million kits at a price of R$34 per unit (around US$8.5 at the time). The test arrived in healthcare facilities in January 2017, however, by which time the number of reported cases of Zika had declined dramatically, and its practical use was immediately hampered by recurrent claims of poor sensitivity (Kameda, 2019).^9^

A slow pace of development, complicated intellectual property negotiations with foreign firms, and the challenge of cross-reactivity with non-Zika antibodies held up several other serological diagnostic platforms. IgM and IgG assays developed by Fiocruz’s Immunology Technology Institute, Bio-Manguinhos, for example, did not become available until April 2019. In the meantime, the only available alternative were the products commercialized by foreign companies, which, in addition to their high cost, presented the problem of having been validated with samples from international travellers. This limited their reliability in Brazil, where ZIKV was typically a secondary flavivirus infection (Felix et al., 2017; Kikuti et al., 2018). The clearest evidence of the lack of diagnostic capacity for antibodies is the absence of large seroprevalence studies. By the time the Ministry of Health lifted the emergency declaration in May 2017, only one extensive serosurvey (in the city of Salvador) had been conducted.^10^

This patchwork of diagnostic options meant that, for the duration of the emergency and beyond, testing was expensive, sporadic and often equivocal. These deficits constrained the ability of public health authorities to gain an accurate picture of the scope and evolution of the epidemic as it unfolded. Disease surveillance relied on passive case detection, and testing was thus limited to the small proportion of the population who sought medical help or otherwise came to the attention of health authorities. Even those groups for whom diagnosis should have been an urgent priority – pregnant women at risk of developing ZIKV-induced congenital abnormalities – had no easy access to testing. It was not unusual for women in some of the hardest-hit areas of the Northeast to have to wait weeks or months to receive the results of a RT-PCR test, if the result came at all. And even when a result was forthcoming, it was often unreliable: In addition to the short viraemic period and the practical difficulty of detecting low quantities of RNA in blood or urine, molecular diagnosis remained beset by a scarcity of high-quality reagents and erratic quality control. An external evaluation conducted in 2017 found an overall risk of false-negative results of 16.7%, while false-positives accounted for 26.7% of total results (Fischer et al., 2018).^11^

In this context, epidemiologists struggled to develop an accurate picture of the prevalence of ZIKV infection, and state authorities had little situational awareness of the evolution of the epidemic. It was readily apparent that cases of microcephaly and other inborn anomalies were disproportionately concentrated in the Northeast, with smaller clusters in Rio de Janeiro and other urban centres, but without extensive seroprevalence studies it was impossible to determine whether the circulation of the virus was similarly restricted to that region (Possas et al., 2017). This uncertainty opened up a space for alternative explanations of the observed ‘microcephaly epidemic’. In the early weeks of the outbreak, theories about the possible influence of (among others) contaminated drinking water, prior immunizations, bovine diarrhoea virus or exposure to the chemical pyriproxyfen (commonly used in Brazil to treat mosquito breeding sites) proliferated in the pages of scientific and popular media, stoking public anxieties and suggesting different courses of public health action.

In parallel, some foreign observers contended that the epidemic of microcephaly could in fact be an artefact of faulty reporting, reflecting simply an overzealous search for newborns with slightly smaller heads (see Butler, 2016). At the root of this particular claim were long-standing doubts about the accuracy of the registration system for birth anomalies in Brazil. Congenital anomalies are notified to the national Live Birth Information System, the *Sistema de Informações Sobre Nascidos Vivos*, but the information recorded in birth certificates is often inadequate, and microcephaly has historically been one of the most frequently underreported malformations. Furthermore, the official threshold of ‘microcephaly’ changed several times in the early weeks of the emergency, before it settled on a head circumference ⩽31.9 cm and ⩽31.5 cm for full-term male and female newborns respectively, in line with WHO recommendations. Despite efforts to harmonize measurements across the country, questions persisted about the reliability of the baseline data against which a spike in the number of cases could be properly determined.

Doubts about the real incidence of microcephaly and other congenital malformations were aggravated by a structural blind-spot in the system: abortion, although illegal, is widespread in Brazil, and exists entirely outside the epidemiological gaze of the state. As the Zika crisis unfolded, it was impossible to know how many women were terminating their pregnancies due to the suspected or confirmed risk of Zika-induced foetal anomalies. Indirect evidence suggested that the number of abortions had increased significantly. Women on Web (WoW), a non-profit organization that provides access to abortion medications through online telemedicine, reported that requests from Brazil had doubled between November 2015 and March 2016 (Aiken et al., 2016). This sort of data had little epidemiological significance, given that the number of Brazilian women who use this service is very small in relation to the estimated half a million illegal abortions carried out annually in Brazil, but it alerted researchers and public health officials to the possibility that the number of cases of congenital anomalies could be much greater than those being registered at birth.

The combination of sporadic data on the prevalence of ZIKV infection in the population and chronic gaps in the reporting of foetal anomalies created a consistently ambiguous epidemiological picture. Throughout 2016 and 2017 researchers produced divergent estimates of the distribution of infection. In a study assessing the evolution of the epidemic during the first year of the emergency, a group of Brazilian epidemiologists argued that the first wave of infection had been largely concentrated in the Northeast, while a second wave, from September 2015 onwards, had had a much broader geographical reach (Oliveira et al., 2017). This study, however, was based on an expansive case definition for ZIKV infection – ‘in view of the sparse availability of confirmatory testing’, researchers were forced to include in their model not only the cases of Zika virus disease directly reported to SINAN, but also those suspected cases of dengue and chikungunya that had been registered with SINAN but lacked confirmatory laboratory diagnosis. In fact, most of the cases used to produce this crucial epidemiological map of the Zika outbreak were reported but ultimately discarded cases of dengue, rather than positively confirmed instances of ZIKV infection.

In a retrospective study published in 2019, an international group of epidemiologists and infectious disease modellers suggested a somewhat different evolution of the epidemic. They argued that the circulation of the virus had been largely restricted to the Northeast, with 94% of an estimated 8.5 million total cases between 1 January 2015 and 23 May 2017 occurring there (Brady et al., 2019: 21). This conclusion contradicted earlier studies that had projected a more widespread distribution of the virus, but its true significance lay in the light it shone on the quality of the data that was produced during the emergency. The authors of the study assumed that ‘a large proportion of Zika and potentially microcephaly cases are likely to have not been reported by routine surveillance’, and were thus driven to rely for their estimates on a combination of educated guesses and heroic modelling work. As far as the true distribution of the virus during the critical period of the emergency is concerned, this has remained the case to this day: Public health intelligence has been limited to an exercise in epidemiological imagination.

## Official diagnosis and its predicaments

Medical diagnosis is an indispensable step in gaining access to civil rights and public services; it is generally a fundamental aspect of what Petryna (2002) calls ‘biological citizenship’, the ability to link particular forms of bodily suffering to the provision of state-sanctioned care and support. The Brazilian Constitution of 1988 enshrined a universal right to health, and the country’s Unified Health System is the largest public healthcare system in the world by number of beneficiaries. Yet in a context of extreme inequality the standard of care varies significantly across socio-economic groups, and is often determined by the ability to afford additional private services. The struggle to make the universal right to health effective beyond the letter of the law thus remains central to the country’s democratic aspirations and social cohesion, an issue that the Zika crisis threw into sharp relief.

The lack of accessible and reliable Zika diagnostics had direct consequences for the ability of Brazilian women and their families to claim the full protection of the state. According to a Directive issued by the Brazilian government in December 2015 and re-stated in March 2016, only children with a ‘confirmed’ diagnosis of congenital Zika syndrome (CZS) were entitled to specialist medical care and support. This care and support was very often insufficient, a fact that has prompted different forms of activism and patient mobilization by the so-called ‘Zika mothers’ or ‘mães de micro’ (Fleischer and Lima, 2020; Scott et al., 2017). Yet the situation was even worse for children who suffered neurodevelopmental impairments but did not receive an official CZS diagnosis, as they had no recognized right to additional care. The narrow scope of patient entitlements made the work of diagnosing CZS particularly crucial and fraught.

The starting point of an official investigation of congenital Zika syndrome was microcephaly, as determined by the threshold of ‘normal’ head circumference. Such a threshold, no matter how precisely set or uniformly measured, excluded by definition children who were born with a head circumference within the normal range but went on to experience alterations in growth and development as a result of exposure to the virus *in utero*. Such a possibility was unknown to clinicians and researchers at the start of the epidemic, and only became apparent as the crisis unfolded and the spectrum of potential symptoms associated with ZIKV infection expanded (Van Der Linden et al., 2016).

From December 2015 onwards, suspected cases of microcephaly – whether detected in utero, at birth, or postpartum – were reported to a Public Health Events Registry (*Registro de Eventos de Saúde Pública*, or RESP) specifically created to track the progression of congenital anomalies possibly linked to ZIKV infection. By the time Brazil lifted its national emergency declaration in May 2017, 13,490 suspected cases of microcephaly or other central nervous system malformations had been reported to the RESP. Of those, only 2653 had been officially confirmed as CZS, and 105 were considered ‘likely’. 5712 cases had been ‘discarded’, and 1784 were ‘excluded’ from the registry (Brasil, Ministério da Saúde, 2017a). The rest were still under investigation. These included cases categorized as ‘inconclusive’, where microcephaly was diagnosed at birth but no follow-up investigation had been undertaken, often because parents refused further examinations.

The criteria for including registered cases of microcephaly in one or another category were a combination of federal definitions and state-level protocols. The category of ‘discarded’, the largest numerically, was semantically hollow – it described cases that had initially ‘met the definition for notification’ but were found upon investigation ‘not to fulfil the definitions of confirmed, probable, inconclusive or excluded/inactive’ (Brasil, Ministério da Saúde, 2017b: 53). ‘Excluded’ cases, in contrast, were those found not to have met the criteria of inclusion in the first place; some were infants who had been born in the first weeks of the crisis, when a more permissive definition of abnormal head circumference had been in use, but the majority were cases that appeared to have been erroneously entered into the registry (duplicates, files with mistyped codes, etc.). Their exclusion was generally presented as a matter of ‘cleaning’ the data by eliminating obviously mistaken entries.

What was the process by which an initial diagnosis of microcephaly was transformed into an official, state-recognized case of CZS, and why was this process so protracted? In the first weeks of the emergency, the Brazilian Ministry of Health issued a protocol according to which all pregnant women suspected of having been infected with Zika should undergo serological and molecular testing (Brasil, Ministério da Saúde, 2015). Given the limited availability of diagnostics, however, laboratory tests ended up playing a limited role in establishing whether a case of microcephaly could be associated with exposure to the virus. The confirmation of CZS relied instead on the identification of structural brain defects in the newborn, which were then linked epidemiologically with a high risk of maternal infection during pregnancy. In the majority of cases, confirmatory evidence of malformations was produced by computerized tomography or cranial ultrasound – magnetic resonance imaging was rarely used, and the technique was generally not available through the Unified Health System.

There is conflicting evidence on how different levels of access to healthcare across regions and socio-economic groups affected the ability to confirm a case of CZS. In their retrospective study of the association between ZIKV infection and microcephaly, Brady et al. (2019) found no evidence of a regional bias in access to confirmatory diagnosis. Other pieces of the puzzle point in a different direction. Diniz and colleagues note, for instance, that the number of ‘discarded’ cases per live births in the state of Alagoas was twice that in the state of Bahia (Diniz, 2017b). Such a huge discrepancy between two proximate Northeastern states suggests two possibilities, not mutually exclusive: that clinical practices were significantly different across state lines and yielded different rates of ‘false’ positives in the initial determination of microcephaly, or that the ability to confirm a presumptive case was unevenly distributed because women in some states had greater access to the means of production of evidence.

Regional and social inequities in the quality of prenatal care in Brazil are well documented, and this includes different levels of access to specialist imaging techniques (Chazan and Faro, 2016). Coverage of ultrasound equipment is uneven, and in rural areas of the Northeast that were heavily affected by the outbreak access to a specialized health care unit often required long and expensive travel (Peiter et al., 2020; see also Viellas et al., 2014). Further discrepancies stem from the highly heterogeneous standards of care available in the private market for antenatal imaging. Brazil has a high density of private ultrasound facilities, but the sector comprises a wide spectrum of providers, from small clinics with poorly trained operators to advanced (and expensive) hospitals equipped with cutting-edge technology and skilled staff (Löwy, 2018). Research into the clinical experience of pregnant women during the Zika emergency suggests that, even when state-of-the-art ultrasonography was available, the interpretation of the evidence and the thoroughness with which a diagnosis of microcephaly was explained were subject to significant disparities across healthcare facilities and socio-economic groups (Carneiro and Fleischer, 2018). Mendes et al. (2020) recount the cases of mothers who first learned of a microcephaly diagnosis when reading their child’s birth certificate, and note the general helplessness they felt in the face of ‘a chronic disease barely understood scientifically’ (p. 3788).

If the scarcity of reliable laboratory diagnosis and inconsistencies in the quality of antenatal care limited diagnostic options for many women, the criminalization of abortion severely constricted the actions they could take upon any knowledge they were able to obtain. In countries where abortion is legal, such as France and French overseas territories, official directives stated that pregnant women thought to have been infected with the virus should be tested for ZIKV antibodies and the presence of the virus itself in blood and urine; if those tests were positive, the women were advised to undergo amniocentesis and frequent ultrasound examinations (Picone et al., 2017). French medical authorities implicitly assumed that, upon receiving a diagnosis of foetal malformations, some women would elect to terminate the pregnancy. Indeed, during the Zika epidemic in French Polynesia in 2013–2014, researchers had initially failed to make the connection between ZIKV infection and microcephaly because many of the women who received a diagnosis of foetal brain abnormalities chose to have an abortion (Cauchemez et al., 2016).

In Brazil and other countries with highly restrictive abortion legislation, the question of what testing was *for* – or more pointedly *not for* – imbued diagnosis with a very different set of connotations. Several studies suggest a radical ambivalence in the position of many Brazilian women vis-à-vis diagnosis and medical knowledge more generally. A reluctance to undergo foetal ultrasonography had been already evident among participants in the first cohort study of pregnant women exposed to the Zika virus. In that study, which started in Rio de Janeiro in September 2015, more than half of the women who tested positive for ZIKV infection declined to undergo imaging studies ‘because the obstetrical facility was too far away or because of fear of possible fetal abnormalities’ (Brasil et al., 2016). These women had been enrolled in a research study and had access to high-quality care in a large metropolitan area; one can only imagine that the ambivalence would be even greater among those with more limited access to clinical resources. In her study of women in rural Paraiba, Diniz notes a distinct disinclination to undergone prenatal testing among women who presented symptoms of ZIKV infection. ‘They choose not to know, because there is nothing they can do: without the right to terminate the pregnancy and with many scientific uncertainties, an early diagnosis is psychological torture’ (Diniz, 2016).

Medical diagnosis was not linked a hopeful clinical pathway – it afforded a predicament, rather than a promissory course of action. For many women, this created a space for what Carneiro and Fleischer call ‘social diagnosis’, a search for ‘more comprehensive existential explanations’ for the birth of a child with severe neurodevelopmental challenges (Carneiro and Fleischer, 2018: 715; see also Brown et al., 2011). This sort of diagnosis implied a shift of emphasis, from a narrow technical determination of the cause of CZS – the identification of the specific disease – towards interpretive frameworks that linked the experience of Zika motherhood to moral values and future opportunities for self-determination. Drawing on interviews with mothers in Recife, Carneiro and Fleischer mention the significance, for some women, of understanding microcephaly as a divinely mandated task and part of their life’s mission. In fact, a recalibration of the significance of medical knowledge has pervaded all aspects of parenting in the wake of Zika. In her study of mothers of children diagnosed with CZS in Salvador, K. Eliza Williamson highlights an intentional decision to *not* anticipate possible outcomes. For many of these women, most of whom were black residents in working class sections of the city, a modicum of hope could only be sustained through ‘a conscious effort to “expect nothing”’ (Williamson, 2018: 685; see also Scott et al., 2018; Williamson, 2020).

The choice not to know, or a deliberate effort not to expect or anticipate, can only be understood in the context of a biomedical landscape that offered limited diagnostic options and few opportunities to act meaningfully on the information they provided. This landscape, however, was not the same for all. Upper- and middle-class women with the resources to access private health services had more options – they were more likely to access laboratory tests in a timely manner and could undergo several antenatal tests, thus increasing the probability of an accurate diagnosis of congenital Zika syndrome earlier in their pregnancy. They could then make a decision on the basis of that knowledge, and if they chose to terminate the pregnancy their decision would likely not come to the attention of the state (Avelino-Silva, 2018; Prado, 2018).

This fundamental divergence goes a long way in explaining the particular social and racial connotations that the Zika epidemic eventually acquired in Brazil. The great majority of children diagnosed with microcephaly were born to poor women of colour. The concentration of cases in the Northeast – where poverty is strongly associated with skin colour – further solidified the view that this was a pathology that affected predominantly poor, non-white people in historically disadvantaged regions of the country (Diniz, 2017a; Freitas et al., 2019, Matos et al., 2018). What was originally deemed a ‘national’ emergency was thus soon disaggregated into socio-economically and racially divergent levels of exposure. On-going cuts to healthcare budgets, and the conservative political turn that began in the summer of 2016, further cemented this trend, making Zika a neglected disease within Brazil itself (Human Rights Watch, 2017).

## Conclusion: Imperfect diagnosis, fragmented global health

Anticipating the legacy of the Zika crisis as it was taking hold, the UK-based epidemiologist Laura Rodrigues made a prescient prediction. ‘I expect’, she wrote, ‘we will teach our students about the production of science using examples from this Public Health Emergency of International Concern for many years to come’ (Rodrigues and Buscato, 2016). Several years after the WHO declared the end of the emergency, it remains doubtful, however, whether the lessons from the Zika emergency have been fully drawn.

The Zika epidemic was understood by international organizations and multilateral agencies as a ‘test case’ for their ability to mount an effective public health response through the acceleration of R&D efforts, following the perceived success of this approach during the 2014–2016 Ebola Virus outbreak in West Africa. As news of a new disease spread, governments and donors promised to invest heavily in new diagnostic and therapeutic tools. As with Ebola, the WHO hoped to leverage its normative authority to act as ‘global convener’ of public, private and philanthropic stakeholders, spurring commercial R&D investment in the absence of a ‘natural’ market for those products (WHO, 2016: 8). While the scale of research undertaken during the Zika emergency period is undeniable – in the months following the PHEIC declaration the world witnessed ‘an explosion of scientific work’ on different aspects of the virus and its pathogenic potential – the output of this effort in terms of novel preventative, therapeutic or diagnostic technologies was less impressive.^12^

The contrast with the experience during the West Africa Ebola virus outbreak suggests that not all global health emergencies are ‘global’ in the same way. The Zika crisis unfolded within a very different geopolitical landscape. Its epicentre was Brazil, a country that combines an advanced infrastructure for the production of biomedical knowledge with a universal health system that struggles to apply that knowledge evenly across regions and socio-economic groups, and where impressive achievements in the control of numerous transmissible diseases coexists with chronic failures to tackle others (Barreto et al., 2011). Brazil is often defined as an ‘intermediary economy’ or a ‘semi-peripheral country,’ but these terms do little more than highlight the general inadequacy of bipolar understandings of the world, and of the binaries that have historically structured the field of global health. After playing a leading role in the fight against pharmaceutical monopolies and pioneering new alliances between an activist state and civil society organizations against AIDS, by the late 2000s Brazil had reverted to ‘a state of triage and a politics of survival’ (Biehl, 2009), precariously balanced between its universalist aspirations and a domestic reality of entrenched and extreme inequality. The 2008 financial crisis and the subsequent economic downturn had only accentuated this contradiction. They revealed Brazil as a country ‘too rich to be considered poor and, at the same time, with no definitive right to be a full part of the so-called first world’ (Cueto and Lopes, 2019: 21).

These were the conditions for a profound misalignment between the R&D efforts sponsored by supranational actors, focused on creating a ‘global’ pipeline of new diagnostics, and a state still committed to finding national – and preferably public – solutions for a domestic health crisis. For much of the emergency period, efforts to tackle the disease in Brazil ran in parallel, but rarely intersected, with those of the international community as represented by WHO. The most visible marker of this separation is the restricted circulation of materials (biological samples in one direction, reagents in the other), but the divide is also evident in the divergent trajectories of diagnostics development. None of the molecular assays listed under the WHO’s emergency use procedure played a significant role in the national effort, while the country sought to develop affordable national alternatives. Brazil’s limited success in this endeavour has less to do with the sort of generic ‘market failure’ that the WHO was hoping to tackle, and much more with insufficient financial and technical resources at the disposal of public health research institutions.

If national capacity was limited, global efforts proved ephemeral. The claim that ‘outbreaks are unsustainable markets’ proved prescient, and that unsustainability became apparent as soon as the number of reported cases of ZIKV infection and congenital malformations began to decline. By the time the WHO lifted its PHEIC in November 2016, there was little in the way of a robust global diagnostics pipeline, and few elements of a longer-term R&D infrastructure were in place. With no significant outbreaks in rich countries, the incentives created by the WHO’s EUAL process proved short-lived.

Global R&D efforts also proved unable to circumvent the ‘elephant in the room’: the divisive politics of abortion and women’s reproductive rights in Brazil and several other of the most directly affected countries. The technical apparatus of international emergency-preparedness and response was unable to address, let alone adjudicate, these disputes and no progress or compromise was forthcoming from the executive bodies of global health institutions. The question of sexual and reproductive health and rights remained fundamental, however, and got to the heart of the matter of diagnosis. Not only did the ban on abortion contribute to a blurry epidemiological picture during the emergency, but it also determined the value (or lack thereof) of diagnostic knowledge for those who were expected to benefit most directly from the new tools, pregnant women exposed to the risk of ZIKV infection. Without a minimum agreement on what diagnosis should be *for* – and *for whom* – international coordination could never be equal to the challenge at hand.

No doubt the overwhelming impact of the ongoing Covid-19 pandemic will reshape our understanding of global health and its history, making our present moment appear as a critical inflection point. Yet the experience of Zika diagnostics suggests that the fault lines that have become glaringly apparent in recent months were already in evidence during the international response to his previous public health emergency. It also shows that ‘emergency R&D,’ as currently conceived, is no substitute for mechanisms that address the geopolitical and socio-economic cleavages that determine whether, and in what manner, public health crises become effectively ‘global.’

## Notes

1 We define as the Zika emergency the period between 11 November 2015 – when the Brazilian Ministry of Health declared a Public Health Emergency of National Importance – and 11 May 2017 – when that declaration was lifted. This encompasses the period (1 February 2016–18 November 2016) when the WHO’s Public Health Emergency of International Concern was in force.

2 Following the Zika outbreak, the pathway was renamed the Emergency Use Listing, or EUL, to address complaints that that ‘A’ in EUAL, signifying ‘assessment’, was often taken to mean ‘authorization’ (see Kelly et al., forthcoming).

3 Remarks offered at the Scientific Consultation on Zika Virus Vaccine Development co-convened by the World Health Organization and the National Institute of Allergy and Infectious Diseases, National Institutes of Health. Rockville, Maryland. 10–11 January 2017.

4 Report presented to the Scientific Advisory Group of the WHO’s R&D Blueprint, February 2017.

5 Presentation by Maria Marinissen (US Department of Health and Human Services) at the 2018 meeting of the Federal Laboratory Consortium for Technology Transfer. Philadelphia, May 2018.

6 Interview, December 2019. Multilateral organizations were not always averse to raising the question of abortion. The Zika Ethics Consultation convened by PAHO in 2016 concluded that women should be able ‘to choose among all relevant reproductive options,’ including termination of pregnancy, given ‘the significant mental anguish about reproductive issues’ that they were experiencing during the outbreak (Pan American Health Organization [PAHO], 2016: 5). The issue was taboo, however, in executive bodies of international organizations where national governments had veto power.

7 Jarbas Barbosa, Anvisa Director President, May 2016. Following this exchange, Anvisa requested a public ‘clarification’ from Fiocruz. In a subsequent press release, Fiocruz declared that Stabeli’s statements had been taken out of context, and that Anvisa represented ‘a firm shield against pressures from the private market, guaranteeing the safety and quality of the products to be used by the population’. See ‘Fiocruz esclarece declarações sobre a Anvisa publicadas pela revista Época.’ Anvisa, 6 June 2016.

8 Interview with researcher at Fiocruz public health laboratory, December 2018.

9 The Ministry of Health eventually abandoned the test due to its unreliability. In February 2019 Anvisa banned Bahiafarma from producing rapid tests for Zika, dengue and chikungunya.

10 This study showed a high rate of ZIKV seroprevalence, particularly among groups of low socio-economic status (Netto et al., 2017). While consistent with clinical evidence and the experience of other arboviruses, the data was limited to the situation in a single city.

11 Seventy-three per cent of the laboratories that took part in the study delivered results with substandard levels of sensitivity and specificity. These shortcomings were not limited to Brazilian laboratories. A similar study conducted in Europe found suboptimal performance in 60% of the surveyed laboratories.

12 Bruce Aylward, WHO Assistant Director-General, quoted in Maurice (2016).
